# PINK1/Parkin Mediated Mitophagy, Ca^2+^ Signalling, and ER–Mitochondria Contacts in Parkinson’s Disease

**DOI:** 10.3390/ijms21051772

**Published:** 2020-03-05

**Authors:** Lucia Barazzuol, Flavia Giamogante, Marisa Brini, Tito Calì

**Affiliations:** 1 Department of Biomedical Sciences, University of Padua, 35131 Padua, Italy; lucia.barazzuol@studenti.unipd.it (L.B.); flavia.giamogante@unipd.it (F.G.); 2 Department of Biology, University of Padua, 35131 Padua, Italy; 3 Padova Neuroscience Center (PNC), University of Padua, 35131 Padua, Italy

**Keywords:** PINK1, Parkin, mitophagy, Ca^2+^, ER–mitochondria tethering

## Abstract

Endoplasmic reticulum (ER)–mitochondria contact sites are critical structures for cellular function. They are implicated in a plethora of cellular processes, including Ca^2+^ signalling and mitophagy, the selective degradation of damaged mitochondria. Phosphatase and tensin homolog (PTEN)-induced kinase (PINK) and Parkin proteins, whose mutations are associated with familial forms of Parkinson’s disease, are two of the best characterized mitophagy players. They accumulate at ER–mitochondria contact sites and modulate organelles crosstalk. Alterations in ER–mitochondria tethering are a common hallmark of many neurodegenerative diseases including Parkinson’s disease. Here, we summarize the current knowledge on the involvement of PINK1 and Parkin at the ER–mitochondria contact sites and their role in the modulation of Ca^2+^ signalling and mitophagy.

## 1. Introduction

Parkinson’s disease (PD) is an incurable chronic progressive neurodegenerative disease affecting nearly 2% of the “over 50” population, with an approximate estimate of more than 6 million cases worldwide [1]. It is characterized by the preferential loss of dopaminergic neurons in the substantia nigra pars compacta, which results in progressive motor system malfunction, accompanied with the accumulation of proteinaceous aggregates, referred to as Lewy bodies (LB), in the remaining neurons of affected individuals [2]. Although the aetiology of PD remains unknown, increasing evidence supports the involvement of both genetic and environmental factors, aging being one of the major risk factors. Among the total Parkinson′s patients, only 5%–10% are familial—they carry heritable, disease-associated monogenic mutations in genes referred to as the *PARK* genes [3]. Genome-wide association studies have established 26 PD risk loci to date and, among the different *PARK* genes, those which encode α-synuclein (*PARK*1/4), Parkin (*PARK*2), PINK1 (phosphatase and tensin homolog (PTEN)-induced kinase 1—*PARK*6), DJ-1 (*PARK*7), LRRK2 (*PARK*8), and ATP13A2 (*PARK*9) have been deeply investigated. Mutations in both LRRK2 and α-synuclein result in autosomal dominant inherited disease, whilst mutations in DJ-1*,* Parkin*,* PINK1, and ATP13A2 proteins give rise to autosomal recessive forms. Many of these proteins are known to participate in the maintenance of mitochondrial or lysosomal functions. Indeed, familial PD patients usually show mitochondrial defects and impairment of the autophagic pathway [4,5], indicating that these two aspects are critical components of PD pathogenesis. In particular, mice defective in autophagy/mitophagy have been shown to recapitulate a series of phenotypes resembling some PD features such as behavioural defects, reduction in coordinated movement, neuronal loss in the cerebral and cerebellar cortices, and accumulation of polyubiquitinated proteins as inclusion bodies [6,7]. To this day, among the best characterized PD proteins are Parkin (an E3 ubiquitin ligase) and PINK1 (a serine/threonine kinase with a mitochondrial targeting sequence), whose mutations are the most common cause of recessive PD. Many studies have highlighted their role in mitochondria clearance via autophagy—a process known as mitophagy [8,9]. More recently, Parkin and PINK1 have been shown to localize at the endoplasmic reticulum (ER)–mitochondria contact sites and to modulate the crosstalk between the two organelles [10]. ER–mitochondria contact sites are crucial structures for cellular function, being involved in a plethora of cellular processes, among which there are Ca^2+^ homeostasis, lipid transfer, mitochondrial metabolism and dynamics, apoptosis, and mitophagy [11]. It is not striking, though, that this interorganellar tethering is found altered in PD, as well as in other neurodegenerative diseases [12], but also in cancer and in metabolic diseases. In light of this, the aim of this review is to summarize the literature concerning the PINK1 and Parkin role as multifunctional players in mitophagy and in ER–mitochondria tethering.

## 2. The PINK1/Parkin Pathway

Macroautophagy (referred hereafter as autophagy) is a genetically programmed process that removes unnecessary or dysfunctional cellular components and recycles them [13]. In this process, firstly, a double-membrane structure, known as the autophagosome, is created to engulf targeted cellular elements and later fuses with lysosomes to form autolysosome, where the enveloped contents are degraded. More than 30 autophagy-related genes (*Atg*) have been identified in yeast, and most of them have mammalian homologues [14]. Initially, autophagy was defined as a non-selective pathway, but it is now widely recognized that there are two different types of autophagy—non-selective and selective. Under nutrient deprivation and during development, the non-selective pathway is mainly activated in order to provide nourishment for cell survival. The selective pathway, instead, is active even in nutrient-rich conditions and plays an important housekeeping function, as it is activated to eliminate dysfunctional organelles, protein aggregates, or intracellular pathogens [15]. Selective autophagy requires specific receptors able to recognize targeted ubiquitinated cargos and to recruit the autophagosome machinery [16]. The most characterized type of selective autophagy is mitophagy, which specifically targets damaged mitochondria to degradation [17]. It is a key cellular process, particularly important in post-mitotic and slow-dividing cells (such as neurons), as it promotes the turnover of mitochondria preventing accumulation of dysfunctional organelles.

Though the crucial role of defective autophagy in neurodegeneration is well established [18], its implication in PD has been particularly investigated. In this respect, Narendra et al. [19,20,21,22] firstly linked PINK1 and Parkin to mitophagy, shedding light into the PINK1/Parkin mitochondrial quality control pathway.

**Figure 1:**
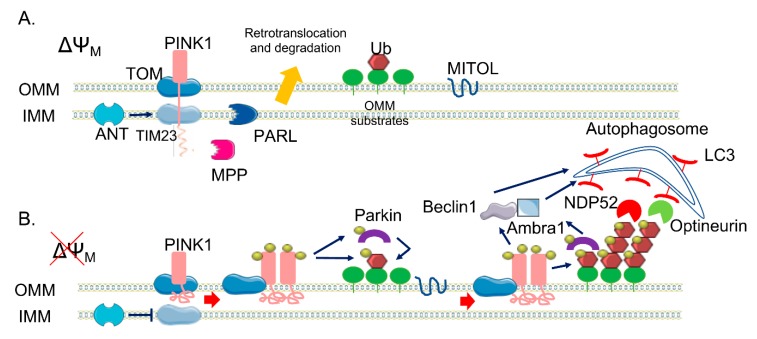
The canonical phosphatase and tensin homolog (PTEN)-induced kinase 1 (PINK1)/Parkin pathway. (**A**) In healthy mitochondria, PINK1 is constitutively imported via translocase of the outer membrane (TOM)/translocase of the inner membrane (TIM)23 complexes to the inner membrane (IMM), cleaved by two proteases (mitochondrial processing peptidase (MPP) and presenilin-associated rhomboid-like (PARL)) and retro-translocated to the cytosol, where it is degraded. (**B**) When ΔΨ_M_ is dissipated, Adenine nucleotide translocator ANT inhibits TIM23-mediated import of PINK1, which is not processed and accumulates on the outer membrane (OMM). Here, a supercomplex is formed, composed by TOM complex subunits and PINK1 homodimers, facilitating PINK1 autophosphorylation and activation. Once activated, PINK1 phosphorylates ubiquitinated substrates on the OMM and thus recruits and phosphorylates Parkin. Phosphorylated Parkin starts to ubiquitylate several proteins on the OMM, which are new substrates of PINK1 phosphorylation—a positive feedback loop is initiated, leading to the coating of damaged mitochondria with phospho-ubiquitin chain, red arrows. The MITOL mitochondrial E3 ubiquitin ligase, seems to be fundamental for the introduction of the initial ubiquitination. Phospho-ubiquitin chains are bound by two mitophagy adaptors, Nuclear domain 10 protein 52 (NDP52) and Optineurin, blue arrows. The two adaptors recruit autophagosomes via Microtubule-associated **protein** 1A/1B-light chain 3 (LC3) binding, allowing the engulfment of dysfunctional mitochondria. In parallel, PINK1 interacts with Beclin-1 and Parkin with Ambra1, further stimulating autophagosome formation, blue arrows.

PINK1 is 581 amino acids long and contains an N-terminal mitochondrial targeting sequence (MTS), a transmembrane domain (TM), a highly conserved serine/threonine kinase domain, and a C-terminal auto-regulatory domain [23]. Under physiological condition, PINK1 levels are quite low because it is rapidly degraded. As a mitochondrial targeted protein, PINK1 is imported into mitochondria through the outer mitochondrial membrane (OMM)-localized TOM (translocase of the outer membrane) complex and the inner mitochondrial membrane (IMM)-localized Tim (translocase of the inner membrane) 23 complex [24]. Translocation of the positively charged MTS through the Tim23 complex is energetically driven by the electrical membrane potential (ΔΨm) across the IMM. After passing through the Tim23 translocase, the N-terminal MTS domain reaches the matrix, where it is cleaved off by the mitochondrial processing peptidase, MPPα/β. This pathway is called the “pre-sequence pathway” (Figure 1). Presenilin-associated rhomboid-like (PARL) is an IMM-resident protease that subsequently cleaves PINK1 in the TM domain between Ala103 and Phe104 [25], producing a truncated 52 kDa protein (the full-length protein is 64 kDa) that is retro-translocated to the cytoplasm and degraded via the N-end rule proteasomal pathway [25]. There are still some controversial data on the precise PINK1 mitochondrial sub-localization—some evidence indicates that PARL may cleave PINK1 at the IMM while the PINK1 catalytic C-terminal domain remains in the cytosol. However, PINK1 has been implicated in the phosphorylation, not always clearly if in a direct or indirect manner, of different proteins that are resident inside mitochondria. Among them there are the High Temperature Requirement Protein A2, also known as HTRA2/Omi protease [26], the chaperone tumor necrosis factor receptor associated protein 1 (TRAP1) [27] in the IMS, and the complex I subunit NDUFA10 (NADH dehydrogenase [ubiquinone] 1 alpha subcomplex subunit 10) located in the IMM [28].

As mentioned before, mitochondrial import requires an electrochemical gradient across mitochondrial membrane, negative on the matrix side, and is inhibited by mitochondrial depolarized agents (e.g., Carbonylcyanure m-chlorophénylhydrazone, CCCP). When translocation within the Tim23 complex halts due to ΔΨm loss, full-length PINK1 is retained in the OMM, with the C-terminal facing the cytosol. Here, PINK1 forms a super-molecular complex (ca 700 kDa) composed of TOM complex subunits and dimeric PINK1 that facilitates its autophosphorylation on Ser228 and Ser402 residues in the kinase domain [29]. Recently, it has been suggested that the ADP/ATP translocase (ANT), which acts as proton gradient-dependent carrier in the IMM, acts as a bioenergetic sensor and is critically required for mitophagy [30]. Indeed, it seems essential for the suppression of TIM23-mediated protein translocation and subsequent stabilization of PINK1 upon mitochondrial depolarization, independently from its ADP/ATP exchange activity. Moreover, PINK1 has been the first identified mono- and poly-ubiquitin kinase [31,32] able to phosphorylate Ub at the conserved Ser65. Ubiquitination is a post-translational modification that typically marks proteins for degradation via proteasomal pathway [33]. However, it can also act as a signal for autophagy [34] and alters substrate activity and localization [35]. Ubiquitination, which is obtained through the covalent bond of ubiquitin to lysine residues or to the N-terminal of the amino group of substrate proteins, is carried out by the sequential action of three enzymes: E1 ubiquitin-activating enzymes, E2 ubiquitin-conjugating enzymes, and E3 ubiquitin ligases. A substrate of the ubiquitination process can be also the ubiquitin (Ub) protein itself, as it contains seven lysine residues and an N-terminal, useful to build up polyubiquitin chain. The most common chain types are K48 and K63 chains, in which multiple ubiquitin molecules are linked in a linear arrangement with the C terminus of one molecule attached to lysine 48 (or 63) of the next. Parkin is a 465 amino acid protein of the RING-between-RING (RBR) family of E3 ubiquitin ligases, so-called because of the presence ring finger-type, or ‘ring’, domains separated by loops responsible for protein-protein interaction, [36,37] with lax substrate specificity [38,39]. It forms multiple types of ubiquitin chains, most frequently K63, K48, K11, and K6 linkages [40]. It is composed of a ubiquitin-like domain (Ubl) at the N-terminus, followed by four zinc-coordinating RING-like domains: RING0, RING1, IBR (in-between-RING fingers), and RING2 [32]. RING domains bind E2 enzymes, though do not participate directly in the catalysis, functioning instead as a scaffold for the transfer of ubiquitin. Structural analysis showed that Parkin RING1 is the only domain similar to a classical RING finger motif, whereas the other three have a completely different structure, suggesting that RING1 is the E2 binding site on Parkin [41,42]. Differently, RING2 contains an active site cysteine (Cys431) that accepts ubiquitin via a thioester bond and then transfers it via an acyl-transfer to the substrate [20,43]. Parkin also contains two flexible linkers, one after the Ubl domain and the other between the IBR and the RING2 domains. The latter is the repressor element of Parkin (REP), named for its role in the regulation of Parkin activity. In normal conditions, Parkin is a cytosolic protein due to its structural auto-inhibitory mechanisms; indeed, the access to the catalytic RING2 domain is blocked by RING0 and, at the same time, the E2 binding site on RING1 is occupied by the Ubl domain and REP linker [44,45]. In addition to the regulation of cellular Parkin levels and activity, the Ubl domain is involved in substrate recognition, binding SH3 and ubiquitin interacting motif (UIM) domains, and associating with proteasomes [46,47,48,49,50]. Parkin itself becomes ubiquitinated by the attachment of K6 ubiquitin chains, which may play a role in its own degradation [40]. Ubiquitin Specific Peptidase 8 (USP8), a deubiquitinating enzyme (DUB), seems to be crucial for Parkin-mediated mitophagy—it preferentially cleaves K6-linked Ub chains from Parkin, a process required for the efficient recruitment of Parkin to depolarized mitochondria. As different studies have identify a role for other DUBs in Parkin-mediated mitophagy, it is becoming clear that, in addition to ubiquitination and phosphorylation, deubiquitination also plays a crucial role in this process [51].

PINK1 acts upstream of Parkin and is required for Parkin activation and recruitment to depolarized mitochondria [21]. In fact, it can phosphorylate Parkin Ubl domain at Ser65 [52], inducing the loss of its auto-inhibitory conformation and the opening of its conformation. The binding of PINK1-phosphorylated Ub to the RING1 domain of Parkin facilitates Parkin phosphorylation by PINK1 [53,54], inducing the further structural rearrangements and the activation of Parkin. Both phospho-Ub and phosphorylation of Ubl domain are required for Parkin full activation, with phospho-Ub serving also as a receptor for Parkin recruitment to mitochondria [55]. Taking into account all of these considerations, a positive feedback loop has been conceived to explain the PINK1/Parkin pathway in mitophagy [8]. The accumulation of PINK1 at the OMM leads to the phosphorylation of low basal levels of both ubiquitin and Parkin present on mitochondria, causing a positive effect on Parkin activity. Activated Parkin attaches Ub protein to OMM proteins, providing also more substrates to PINK1 phosphorylation, amplifying Parkin recruitment and activation. As result of this mechanism, dysfunctional mitochondria can be coated with phospho-ub chains. This amplifying positive feedback explains how high levels of Parkin can be recruited from the cytoplasm to the mitochondria membrane by low endogenous level of PINK1. Recent data also show the implication of the mitochondrial ubiquitin ligase MITOL/MARCH5, which belongs to the membrane-associated RING-CH E3 ubiquitin ligase (MARCH) family (also called MARCH5), one of the three mitochondria-localized ubiquitin ligases (E3s) identified thus far, in providing initial substrates to PINK1 [56]. It seems to act by controlling mitochondrial dynamics through the regulation of the mitochondrial fission/fusion factors such as Dynamin-1-like protein is a GTPase (Drp1) or Mitochondrial fission 1 protein (Fis1) and mitofusin 2 (Mfn2), respectively. In addition, it has been recently observed that a reduction of MITOL levels delays Parkin recruitment to depolarized mitochondria and decreases the efficiency of Parkin-mediated mitochondrial ubiquitination; conversely, its overexpression results in the opposite effect, suggesting that MITOL may introduce the initial “seed” for ubiquitination, promoting rapid Parkin recruitment at the onset of mitophagy (Figure 1).

The mechanism underlying the further degradation of Ub-primed mitochondria is still unclear, but it has been demonstrated that the PINK1/Parkin pathway is implicated also in the phase of the autophagy clearance of damaged mitochondria in several ways. Indeed, it promotes the fragmentation of mitochondrial network, allowing mitochondria to be taken up by autophagosome; it modulates the mitochondrial motility, in order to stop their movement; and it directly recruits the autophagic machinery to dysfunctional mitochondria [57]. PINK1 and Parkin, in fact, modify a wide range of substrate proteins in the OMM, mediating their clearance.

Interestingly, 36 OMM substrates of Parkin have been identified with high confidence [39], suggesting that no specific substrate is required for ubiquitin signalling of mitophagy. However, the best characterized targets are mitofusins 1 and 2 (Mfns 1/2) [58] and voltage-dependent-activated channel 1 (VDAC1) [22]. Mfns are involved in mitochondrial fusion and both Mfn2 and VDAC1 are involved in ER–mitochondrial tethering. As for Mfn2, the pool located on the ER membranes can form hetero- or homo-dimers in trans with mitochondrial mitofusins (i.e., Mfn1 or Mfn2) to fine tune the mitochondria-associated membrane (MAM)-dependent functions [59]. Although it is widely accepted that this protein is a major regulator of the mitochondria–ER interface, the exact role played at the mitochondria–ER contacts is still unclear. In fact, Mfn2 has been shown to have opposite effects in this interorganellar crosstalk [60,61], a fact that could be due to different distance ranges of contacts occurring at the ER–mitochondria interface [62]. As for VDAC1, it interacts with the ER Ca^2+^ channel inositol 1,4,5-trisphosphate receptor (IP_3_R) via the mitochondrial chaperone glucose-regulated protein 75 (Grp75) [63]. This complex seems to be essential for coupling Ca^2+^ transfer between ER and mitochondria [64]. Interestingly, very recently DJ-1 protein has been shown to take part of the VDAC1-IP_3_R_Grp75 complex and be essential for ER-mitochondria tethering [65].

The ubiquitination of both Mfns and VDAC1 and their subsequent proteasomal degradation facilitates the fragmentation of the mitochondrial network, and thus mitochondrial engulfment by autophagosomes. In addition, p97, an AAA+ ATPase, accumulates on mitochondria in a Parkin-dependent manner and promotes the degradation of OMM ubiquitylated proteins [66], leading to the rupture of OMM [67]. A recent study by McLelland and colleagues suggests that Parkin/PINK1 activation catalyses a rapid burst of Mfn2 phosphoubiquitination to trigger p97-dependent disassembly of Mfn2 complexes from the outer mitochondrial membrane, dissociating mitochondria from the ER. This promotes the availability of other Parkin substrates such as VDAC1, thus facilitating mitophagy [68]. Other substrates of the PINK1/Parkin pathway are Miro proteins (1/2), which are components of the primary motor/adaptor complex that anchor kinesin to the mitochondrial surface. Their degradation leads to the blockage of mitochondrial movement, promoting the segregation of dysfunctional mitochondria [69]. In addition, a recent paper by Safiulina et al. [70] suggested a role for Miro1 in Parkin recruitment to damaged mitochondria. In fact, in rat primary cortical neurons, Miro1 seems to be able to interact with Parkin also in the absence of PINK1 accumulation at the OMM and in basal conditions, suggesting a role for Miro1 as a mitochondrial docking site for the recruitment of Parkin from the cytosol. As mentioned above, other proteins have been identified as PINK1/Parkin substrates, but whether their ubiquitination could have a regulatory function rather than a degradative role remains unclear. Interestingly, recent studies have reported proteasome-independent-mediated ubiquitination by Parkin, which does not result in degradative ubiquitination but in fine tuning protein–protein association [71]. The PINK1/Parkin pathway not only primes damaged mitochondria by ubiquitination, but also promotes the induction of mitophagy. Two autophagy adaptors, namely, NDP52 and Optineurin [72,73], are recruited by phospho-Ub to dysfunctional mitochondria, where in turn they recruit components of the autophagic pathway to initiate mitophagy. Moreover, Parkin interacts with Ambra1 [74], a positive regulator of Beclin-1-dependent autophagy. Indeed, Ambra1 locally stimulates the activity of the class III Phosphoinositide 3-kinase (PI3K) complex, essential for the formation of new phagophores. PINK1 was also found to directly bind Beclin1 and to be required for the nucleation of the phagophores and the omegasome (the precursor of autophagosomes) generation [75]. The relative importance of mono- or polyubiquitinated chains on OMM substrates is still unknown, but monoubiquitin targeted to mitochondria is not sufficient to recruit Parkin, thus suggesting a possible specific role for polyubiquitin chains [76]. One last point to be mentioned is the role of phosphatase and tensin homolog (PTEN) itself, which seems to be directly involved in the modulation of mitophagy-related functions. Indeed a direct regulation of mitophagy through the promotion of Parkin recruitment to damaged mitochondria has been recently proposed for the first PTEN isoform identified so far, i.e., PTENα [77], whereas, interestingly, a newly identified long isoform of PTEN (PTEN-L) has been shown to act as a negative regulator of mitophagy by preventing Parkin mitochondrial translocation and inhibiting its E3 ligase activity [78]. Noteworthy, a fraction of PTEN has also been found to localize at the ER membrane and the MAMs and to regulate ER/mitochondria Ca^2+^ transfer [79].

## 3. PINK1, Parkin, and Ca^2+^

PINK1 and Parkin, in addition to their role in the execution of mitophagy, have been shown to regulate a number of different mitochondria-related activities, such as biogenesis [80,81], integrity [82,83,84], respiration [85,86], and Ca^2+^ homeostasis [87,88]. Ca^2+^ homeostasis dysregulation is a key hallmark in many neurodegenerative diseases; in this respect, the role of PINK1 and Parkin in modulating this process has started to be investigated. The first evidence on their involvement in Ca^2+^ handling arose from the finding that the expression of mutant, but not of wild-type, PINK1 exacerbated the mitochondrial defects observed in a cellular model of PD expressing mutated A53T α-syn [87]. The defects, comprising the loss of Δ*Ψ*_m_, the increase of mitochondrial size with loss of cristae, and the reduction of ATP levels, were partially recovered by treatment with cyclosporine A, a drug that desensitize the mitochondrial permeability transition pore opening, and fully rescued by the inhibitor of the mitochondrial Ca^2+^ uniporter (MCU) ruthenium red. These findings suggested a role for excessive mitochondrial Ca^2+^ uptake in the mechanism of mitochondrial dysfunction in PD. It should be mentioned, however, that PINK1 overexpression might induce its mitochondrial accumulation and activation of mitophagy in the absence of other exogenous insults, thus potentially influencing the interpretation of some results [89]. Nonetheless, data obtained by subsequent works are controversial. On one hand, Ghandi et al. [90] suggested that PINK1 regulated Ca^2+^ efflux from the mitochondria via the mitochondrial Na^+^/Ca^2+^ exchanger. As a consequence, PINK1 deficiency caused mitochondrial accumulation of Ca^2+^, resulting in mitochondrial Ca^2+^ overload. On the other hand, Heeman et al. [91] reported that PINK1 depletion impaired mitochondrial Ca^2+^ uptake, as it impacted on Δ*Ψ*_m_, and consequently ATP production. Recently, PINK1 was found to directly phosphorylate the leucine zipper-EF-hand-containing transmembrane protein 1 (LETM1), a mitochondrial inner membrane protein, proposed as mediating mitochondrial proton-dependent Ca^2+^ exchange [92,93,94,95]. Knockdown of LETM1 compromised the rate of mitochondrial Ca^2+^ uptake and extrusion, leading to defects in mitochondrial bioenergetics, metabolic signalling, and sensitization to cell death, especially in neurons. PINK1-mediated phosphorylation of LETM1 appeared crucial for its activity on mitochondrial Ca^2+^ regulation and for neuronal survival. Interestingly, Akundi et al. [88] suggested that PINK1 ablation enhanced the sensitivity to Ca^2+^-induced mitochondrial permeability transition, which was responsible for increased cell death susceptibility and consequently to a reduction of dopamine levels due to dopaminergic neurons loss. Recently, it has been shown that the degeneration of dopaminergic neurons observed in a model of PINK1−/− zebrafish can be rescued by the selective inhibition of MCU [96], strongly reinforcing the hypothesis that the mitochondrial Ca^2+^ uptake pathway could be directly involved in the neuronal degeneration process. Further studies are required to clarify the molecular targets of PINK1 action.

**Figure 2:**
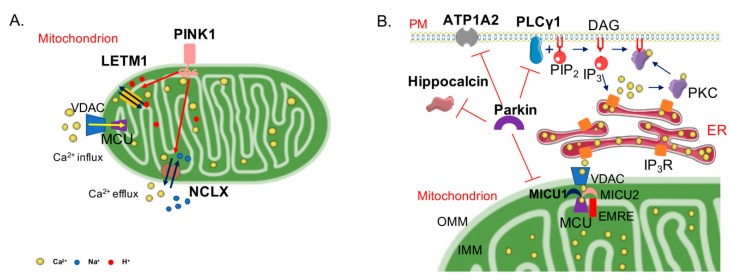
PINK1, Parkin, and Ca^2+^. (**A**) PINK1 is involved in mitochondrial Ca^2+^ handling. In fact, its depletion leads to mitochondrial Ca^2+^ overload. The mechanisms underlying this PINK1 function are not clear, but it has been suggested as being a possible, controversial PINK1-dependent regulation of mitochondrial Ca^2+^ influx via the modulation of Ca^2+^ uptake or efflux via the modulation of the mitochondrial Na^+^/Ca^2+^ exchanger (NCLX). PINK1 also phosphorylates the leucine zipper-EF-hand-containing transmembrane protein 1 (LETM1) by increasing its activity. (B) Parkin involvement in Ca^2+^ homeostasis is more characterized. It regulates mitochondrial Ca^2+^ uptake protein 1 MICU1 turnover, and indirectly also that of mitochondrial Ca^2+^ uptake protein 2 (MICU2), whose stability depends on MICU1. MICU1 and 2 are the positive and negative regulators of the mitochondrial uniporter pore subunit (MCU), respectively. By decreasing MICU levels, Parkin is able to regulate mitochondrial Ca^2+^ handling. Parkin also interacts with and ubiquitylate phospholipase Cγ1 (PLCγ1), responsible for the generation of the second messenger inositol 1,4,5 trisphosphate (IP_3_) and diacylglycerol (DAG). Parkin deficiency leads to a PLC-dependent increase of intracellular Ca^2+^ levels, possibly due to excessive activation of PLCγ1. Two additional Parkin substrates have been recently identified: the ATPase Na^+^/K^+^ transporting subunit alpha 2 (ATP1A2), possibly involved in modulating Ca^2+^ dynamics, and Hippocalcin, a Ca^2+^ sensor that can also regulate voltage-dependent Ca^2+^ channels.

As for Parkin, it has been shown that its overexpression in HeLa or neuroblastoma SY-SY5Y cells enhances mitochondrial Ca^2+^ transients generated upon stimulation with a Ca^2+^ mobilizing agent and sustains ATP production [97]. Interestingly, the Parkin Ubl domain is essential for this action, as the overexpression of a ΔUbl Parkin mutant failed to produce the same effects. The importance of Parkin ubiquitin-like domain was also highlighted in a study where the role of Parkin in the regulation of the turnover of the different components of the MCU complex was investigated (Figure 2).

MCU is a Ca^2+^-selective channel of the inner mitochondrial membrane (IMM) composed by pore-forming and regulatory subunits [98]. The pore-forming subunits are MCU, its dominant-negative paralog MCUb, and the Essential MCU REgulator EMRE, whereas the regulatory subunits are mitochondrial Ca^2+^ uptake protein 1 (MICU1), a positive regulator, and mitochondrial Ca^2+^ uptake protein 2 (MICU2), a negative regulator. An additional positive regulator, MICU3, is highly expressed in neuronal cells. MICU1 and 2 work as a dimer and are located in the mitochondrial intermembranes space; they both possess two EF-hand domains, which account for the regulation of MCU opening in a Ca^2+^-dependent way [99,100,101]. Indeed, MICU1 stimulates MCU at high [Ca^2+^], allowing the rapid Ca^2+^ transport in the mitochondrial matrix when required. Conversely, MICU2, by inhibiting MCU activity at low [Ca^2+^], counteracts the excessive Ca^2+^ accumulation under conditions of increased driving force, thus protecting from the processes of Ca^2+^ cycling and matrix overload. In basal conditions, Parkin was found to ubiquitylate MICU1, which is rapidly degraded via the proteasome system. In this way, Parkin indirectly also controls MICU2 levels, as MICU2 stability depends on MICU1 [102]. Even in this case, the Parkin Ubl domain is important for this action because the expression of a ΔUbl Parkin mutant has no effect on MICU1 turnover.

Interestingly, Parkin was found to also participate in the regulation of intracellular Ca^2+^ concentration ([Ca^2+^]i) by interacting with and ubiquitylating phospholipase Cγ1 (PLCγ1) [103]. PLC signalling not only regulates the release of ER Ca^2+^, but also drives to the formation of diacylglycerol (DAG), which results in the activation of protein kinase C (PKC). Parkin deficiency leads to an increase of [Ca^2+^]_i_ levels due to enhanced PLC activity and, as a consequence, makes the cells more vulnerable to neurotoxins. Interestingly, the ATPase Na^+^/K^+^ transporting subunit alpha 2 (ATP1A2) and Hippocalcin, among others, have been recently identified as novel Parkin substrates in a ubiquitylome survey from the brains of aged Parkin-knockout (KO) mice [104]. Little is known on ATP1A2, though its deficiency leads to impaired neuronal activity in neurons [105] and can affect Ca^2+^ homeostasis by modulating Ca^2+^ transients and sarcoplasmic reticulum Ca^2+^ release [106,107]. As for Hippocalcin, it is a Ca^2+^ sensor that modulates G-protein-coupled receptor signals and second messenger cascades, and it has also been reported that it can regulate voltage-dependent Ca^2+^ channels [108].

Altogether, these findings indicate the possibility that the absence of Parkin could impact on the Ca^2+^ -dependent excitability of neuronal cells.

## 4. PINK1, Parkin, and ER–Mitochondria Cross-Talk

**Figure 3:**
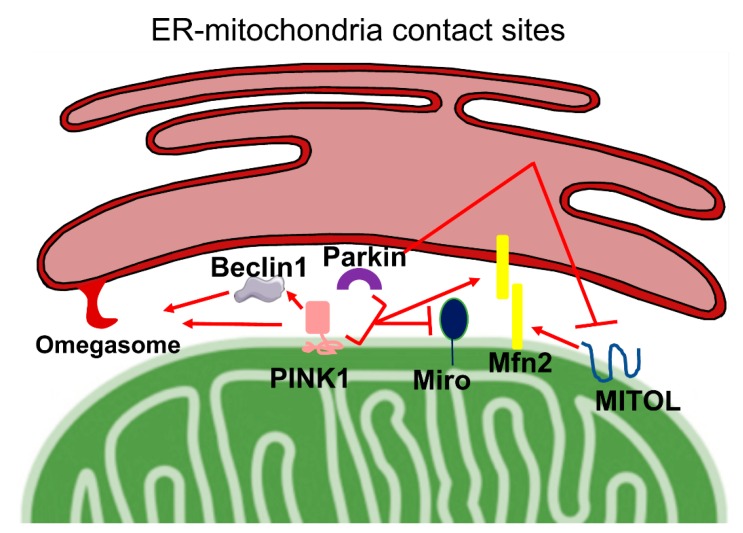
PINK1, Parkin, and endoplasmic reticulum (ER)–mitochondria tethering. PINK1 and Parkin are found enriched at ER–mitochondria contact sites. Upon treatment with CCCP, PINK1 localization is markedly increased in the ER–mitochondria interface, where it recruits Beclin-1, a pro-autophagic protein. ER–mitochondria juxtaposition and omegasome formation (a process that occurs at ER–mitochondrial contact sites) are enhanced upon PINK1-mediated Beclin-1 recruitment, red arrows. Miro1 protein, an OMM Rho GTPase 1, which tethers mitochondria to microtubules and regulates their movement, is a PINK1 substrate, red arrow.

In the last decades, inter-organelle communication has received a great deal of attention by the scientific community, as their dynamic nature appears to influence cell physiology and viability. In particular, the contact sites that the endoplasmic reticulum (ER) forms with mitochondria, and that occur at the so-called mitochondria-associated membranes (MAMs), have emerged as a complex hub that is fundamental for a variety of cellular processes, among which include Ca^2+^ homeostasis, lipid transfer, mitochondrial metabolism and dynamics, apoptosis, and autophagy [11]. In light of this, it is not striking that ER–mitochondria communication is found altered under pathological conditions such as cancer, and metabolic and neurodegenerative disease [12,109,110]. The maintenance of a correct tethering between the ER and mitochondria is achieved through the physical interaction of several cytosolic and integral membrane proteins, whose modulation influences the communication between the two organelles. Although several of the MAMs’ tether and modulator functions have been already established, a growing number of new players have been found at ER–mitochondria contact sites, but their involvement in this communication is still unclear. Interestingly for this review, several proteins whose alterations are linked to the onset of familiar cases of Parkinson’s disease have been localized at the MAMs, consistently with the fact that altered ER–mitochondria communication is a common hallmark of PD. In particular, DJ-1 [111], α-synuclein [112,113], PINK1, and Parkin have been demonstrated to be present at the ER–mitochondria interface [10]. Moreover, PINK1 and Parkin interact with several proteins associated with the MAMs, such as VDACs and Mfn2, strengthening the hypothesis for their potential role as modulators of the ER–mitochondria communication. The role of PINK1 and Parkin on ER–mitochondrial contact sites has been investigated in very few papers, which we attempt to summarize hereafter (Figure 3).

PINK1 has been found to be present at the MAMs in basal conditions and the cell treatment, with CCCP further increasing its localization at this subcellular fraction where it promotes mitophagy. Upon CCCP incubation, PINK1 could also enhance ER–mitochondria tethering, acting together with Beclin-1 that is re-localized at the MAMs in a PINK1-dependent manner [10]. Beclin-1 directly interacts with PINK1, although it is not phosphorylated by it, and is involved in the final step of mitophagy, allowing the nucleation of phagophore and the generation of omegasomes, i.e., precursors of autophagosomes, but could also participate in the modulation of ER–mitochondria tethering. Both PINK1 and Beclin1 silencing has been shown to impair the formation of omegasomes, a process that has been suggested to occur at ER–mitochondrial contact sites. The PINK1 influence on ER–mitochondria tethering could suggest a novel intriguing role for PINK1 in promoting mitophagy.

Interestingly, the silencing of PINK1 in M17 dopaminergic cells caused a reduction in the number of ER–mitochondria contact sites and an increase in their distance [114].

The OMM Rho GTPase 1 (Miro1), as mentioned above, is a target of both PINK1 and Parkin. It tethers mitochondria to microtubules via the binding to Milton protein and regulates their trafficking to defined subcellular localizations. Miro1 possesses two EF-hands motifs, through which it regulates mitochondrial movements in a Ca^2+^-dependent manner. Activation of the PINK1/Parkin mitophagy pathway dramatically reduces Miro1 level, leading to mitochondrial motility dysfunction. A recent study reports that Miro1 silencing in MEF cells reduces the ER–mitochondria tethering, thus suggesting its possible role in the regulation of the apposition of these two organelles. Under stress conditions, PINK1, by targeting Miro1 to degradation through its phosphorylation, could indirectly influence ER–mitochondria tethering, although these data seem not to be in line with the previous findings [69,115]. It is important to mention that Grossman and colleagues have recently described for the first time two heterozygous mutations in the Miro1 encoding gene *RHOT1* in two PD patients. In patient-derived fibroblast harbouring both the discovered mutations a decrease in ER–mitochondria tethering was observed, which consequently caused impaired cellular Ca^2+^ homeostasis and increased Ca^2+^-induced mitochondrial fragmentation, ultimately inducing mitophagy [116].

Differently to PINK1, Parkin involvement on ER–mitochondria tethering has been more thoroughly characterized. As PINK1, Parkin has been shown to accumulate at the MAMs [117], both upon treatment of the cells with CCCP, i.e., upon mitophagy stimuli, and in neurons following glutamate-induced excitotoxicity, supporting a role for Parkin in ER–mitochondria crosstalk not only during mitophagy. In agreement with this possibility, Parkin overexpression in HeLa cells significantly increased the ER–mitochondria contact sites and resulted in the enhancement of ER–mitochondrial Ca^2+^ transfer and ATP production [97]. Although Parkin overexpression has been found to increase ER–mitochondria tethering and its silencing to have the opposite effect in HeLa and dopaminergic SHSY5Y cells, contrasting data were reported in primary cells from patients with *PARK2* mutations and from PARK2 KO mice [117,118,119]. The loss of Parkin in these models resulted either in a greater proximity between the ER and mitochondria [118], also observed in *Drosophila* models [120], or a decreased ER–mitochondria association [119]. Regarding the possible mechanism of Parkin effects on ER–mitochondrial tethering, it has been shown that it is dependent on Parkin-mediated Mfn2 ubiquitination, as non-ubiquitinatable Mfn2 mutant fails to restore ER–mitochondria physical and functional interaction [119]. In support of this idea, Mfn2 downregulation has been shown to restore Ca^2+^ transients in cells with loss of Parkin due to PARK2 mutations [118].

Parkin acts on Mfn2 also via MITOL. Indeed, a recent study has reported that Parkin-dependent ubiquitination of MITOL is responsible for its peroxisomal translocation upon mitophagy induction [121]. Considering that that MITOL has been demonstrated to regulate ER tethering to mitochondria by activating Mfn2 via K192 ubiquitination [122], Parkin action on MITOL redistribution could be an indirect way to modulate ER–mitochondria cross-talk.

## 5. Conclusions

It is well recognized that mitochondrial dysfunction and Ca^2+^ homeostasis dysregulation are both involved in PD pathogenesis, although the exact mechanisms leading to the disease onset are still unclear. It is also very clear that the PD-related proteins PINK1 and Parkin are not only multiplayer actors in the process of mitochondria quality control, but also in the regulation of mitochondria-related processes, even in the absence of mitochondrial damage. As a consequence, the deep understanding of their multitasking roles is essential. In particular, the characterization of the molecular details at the basis of their action in the modulation of ER–mitochondria tethering is important, not only to understand the link between mitochondrial dysfunction and Ca^2+^ dys-homeostasis, but also to discover novel functions of these two proteins, as ER-mitochondria communication is involved in a great plethora of cellular processes.
